# Exploring the Effects of Dietary, Exercise, and Combined Lifestyle Interventions in the Prevention and Management of Gestational Diabetes Mellitus: A Narrative Review

**DOI:** 10.3390/healthcare14091149

**Published:** 2026-04-24

**Authors:** Lujayn Altahan, Jasna Twynstra, Jamie A. Seabrook, Michelle F. Mottola

**Affiliations:** 1 Brescia School of Food and Nutritional Sciences, Faculty of Health Sciences, Western University, London, ON N6G 2V4, Canada; laltahan@uwo.ca (L.A.); jjunuzo@uwo.ca (J.T.); jseabro2@uwo.ca (J.A.S.); 2 Children’s Health Research Institute, London, ON N6C 2V5, Canada; 3 Department of Paediatrics, Western University, London, ON N6A 5W9, Canada; 4 Department of Epidemiology and Biostatistics, Western University, London, ON N6G 2M1, Canada; 5 Lawson Research Institute, London, ON N6A 4V2, Canada; 6 London Health Sciences Centre Research Institute, London, ON N6A 5W9, Canada; 7 R Samuel McLaughlin Foundation—Exercise and Pregnancy Laboratory, Western University, London, ON N6A 3K7, Canada; 8 School of Kinesiology, Faculty of Health Sciences, Western University, London, ON N6A 3K7, Canada; 9 Department of Anatomy & Cell Biology, Schulich School of Medicine & Dentistry, Western University, London, ON N6A 3K7, Canada

**Keywords:** gestational diabetes, combined lifestyle interventions, nutrition interventions, exercise interventions, cultural sensitivity, religious sensitivity

## Abstract

**Objectives:** The objectives of this review are to explore the effects of various nutrition and exercise lifestyle interventions on pregnancy outcomes in individuals with, or at risk of, gestational diabetes mellitus (GDM), as well as to examine whether interventions that are culturally and/or religiously sensitive influence clinical and behavioural outcomes. **Methods:** This study was conducted as a narrative review. PRISMA was used solely as a reporting guide to enhance transparency in the search and study selection process. PubMed/MEDLINE, CINAHL, and Scopus were searched for studies published up to November 2025. Intervention-based studies evaluating nutrition, physical activity, or combined lifestyle interventions targeting either GDM incidence, insulin use, or glycemic outcomes were included. Forty-three studies met eligibility criteria. Study designs consisted primarily of randomized controlled trials (RCTs) with one case–control and one quasi-experimental design trial. **Results:** Combined lifestyle interventions generally showed the most consistent improvements in glycemic control; however, findings were not uniform across all studies, and reporting on insulin outcomes was limited. The Mediterranean, low-glycemic index (LGI) and DASH diets, along with supervised, prenatal exercise programs with low–moderate intensity, delivered at least three times per week, were effective in managing GDM. Regarding culturally or religiously sensitive interventions, only one study was identified. **Conclusions:** Lifestyle interventions may improve glycemic outcomes in GDM; however, further high-quality research is needed, particularly studies incorporating culturally and religiously sensitive approaches and improved reporting of insulin-related outcomes.

## 1. Introduction

Gestational diabetes mellitus (GDM) is one of the most common pregnancy complications, characterized by hyperglycemia that develops or is first recognized during pregnancy [1]. Current estimates suggest that up to 20% of pregnancies worldwide are affected by hyperglycemia in pregnancy, the majority attributable to GDM [2]. GDM is associated with an elevated risk of developing type 2 diabetes [3] and cardiovascular disease later in life [4]. Furthermore, the fetus is at heightened risk for potential complications, including stillbirth, preterm birth, macrosomia, neonatal jaundice, and neonatal hypoglycemia [5]. Similarly, children born to mothers with GDM are more likely to develop cardiovascular complications [6], obesity [7], and type 2 diabetes in the long term [8]. Established risk factors for GDM include pre-pregnancy overweight and obesity, excessive gestational weight gain [9], advanced maternal age [10], and pre-existing insulin resistance [11]. Marked ethnic disparities have also been observed, with higher risk among women of South Asian, Middle Eastern, East Asian [12], African American, Hispanic, and Latin American backgrounds [13].

Pregnancy is a naturally occurring insulin-resistant state. As gestation progresses, maternal tissues become less responsive to insulin so that more glucose remains available for placental transfer to the fetus. This physiologic insulin resistance is largely driven by placental and pregnancy hormones, as well as changes in maternal adiposity and inflammatory mediators. In most pregnancies, pancreatic beta cells adapt by increasing mass and insulin secretory capacity, maintaining near-normal blood glucose concentrations (BGC) despite rising resistance. GDM arises when this adaptive beta cell compensation is inadequate for the degree of pregnancy-induced insulin resistance, resulting in hyperglycemia that is first identified during pregnancy [14].

Given that pregnancy represents a dynamic metabolic state characterized by progressive insulin resistance, nutritional modification is a cornerstone strategy for both the prevention and clinical management of GDM [15]. Medical nutrition therapy (MNT) aims to decrease the risk of developing GDM or to reduce maternal and fetal complications related to GDM. This approach focuses on optimizing maternal glycemic control through nutrition, specifically focusing on the macronutrient composition of the diet [16]. Carbohydrate intake has the most immediate influence on postprandial BGC. Low-glycemic index (LGI) carbohydrate patterns are associated with improved fasting and postprandial glucose control [17], as well as a reduced risk of large-for-gestational-age infants [18] compared with standardized dietary approaches. The glycemic index (GI) is a scale that is used to measure how quickly a carbohydrate-containing food raises BGC post-consumption. High-glycemic index (HGI) foods raise blood glucose concentrations rapidly, whereas low GI foods raise BGC more slowly and steadily [19]. LGI carbohydrates, such as whole-grains, fruits, legumes, and nuts, are associated with improved maternal glucose regulation, whereas refined carbohydrates and low-fiber dietary patterns are linked with increased insulin resistance and greater GDM risk [16,19]. Protein sources similarly influence metabolic outcomes. Higher intakes of animal-based protein, particularly red and processed meats, have been associated with an elevated risk of developing GDM, while plant-based proteins such as soy, legumes, and nuts may improve glycemic control and reduce cardiometabolic risk in affected pregnancies [16,19]. Fat intake also requires careful consideration, as pregnancy naturally induces changes in lipid metabolism, and individuals with GDM demonstrate altered maternal and fetal handling of long-chain polyunsaturated fatty acids. Increasing intake of foods high in omega-3 fatty acids has been shown to improve fasting glucose, insulin resistance, and lipid profiles among individuals with GDM, highlighting the importance of prioritizing healthy fat sources during pregnancy [16,19].

Physical activity represents a key adjunct strategy for both the prevention and management of GDM, given its potent effects on insulin sensitivity and glycemic control [20,21]. Regular physical activity enhances insulin sensitivity by increasing skeletal muscle glucose uptake through both insulin-dependent and insulin-independent pathways [22], thereby helping to counteract the progressive insulin resistance characteristic of pregnancy [23]. Because skeletal muscle contraction stimulates GLUT-4 translocation even when insulin signalling is impaired, exercise offers a powerful non-pharmacological strategy for lowering maternal BGC and reducing the likelihood of excessive fetal growth and other pregnancy complications. When combined with MNT, structured physical activity provides a synergistic approach to mitigating pregnancy-induced insulin resistance and improving maternal–fetal metabolic outcomes [24].

Given the central influence of exercise and nutrition on maternal glycemia and pregnancy outcomes, nutrition and exercise form key elements of lifestyle interventions. This narrative review will first explore the effects of various nutrition and exercise lifestyle interventions on pregnancy outcomes in individuals with, or at risk of, GDM. While previous reviews have provided valuable insights, they have typically focused on either the prevention or management of GDM in isolation [25,26]. One review was identified that evaluated both GDM prevention and management intervention studies; however, it synthesized secondary evidence from other reviews [27]. Accordingly, this review will integrate evidence from original research studies to provide a more comprehensive understanding of how lifestyle interventions perform across the continuum of care. Additionally, this review will also examine whether culturally and/or religiously sensitive interventions influence clinical and behavioural outcomes, as this has not yet been specifically addressed in the existing reviews on GDM prevention or management. Culturally and/or religiously sensitive interventions are those that integrate relevant cultural values, dietary practices, and faith-based norms into care delivery [28,29].

## 2. Methods

### 2.1. Study Design

This study was conducted as a narrative review. As such, the review does not adhere to the full methodological requirements of a systematic review or strictly follow PRISMA guidelines. PRISMA was used solely as a reporting guide to enhance transparency and methodological clarity, with a PRISMA-style flow diagram outlining the study identification, screening and selection process.

### 2.2. Search Strategy

A literature search was conducted to address the objectives of the review. A health sciences librarian was consulted during the development of the search strategy to assist in identifying appropriate databases and refining the search terminology. The complete search strategy underwent formal peer review to ensure methodological rigour, accuracy, and alignment with the study objectives.

The following databases were searched: PubMed/Medline, CINAHL, and Scopus. Trial registries, the grey literature, and reference lists were not considered. The final search, which was conducted on 3 December 2025, captured articles published up to November 2025. Language restrictions were applied during the search. All eligible studies were analysed in the English language, either because they were originally published in English or because they were translated into English. Any article not available in English was excluded.

The PubMed search was conducted using the following search terminology: (“gestational diabetes mellitus” [MeSH] OR “gestational diabetes” [tiab] OR GDM[tiab]) AND (intervention*[tiab] OR “intervention studies” [MeSH] OR trial[tiab] OR “lifestyle intervention” [tiab]) AND (diet[tiab] OR nutrition[tiab] OR “dietary intervention” [tiab] OR “physical activity” [tiab] OR exercise[tiab] OR “exercise therapy” [MeSH] OR “physical activity intervention” [tiab] OR “combined lifestyle” [tiab]). This terminology was slightly modified when searching Scopus and CINAHL to suit the unique style of the databases.

### 2.3. Inclusion and Exclusion Criteria

Studies were selected based on predefined inclusion and exclusion criteria aligned with the objectives of the review. Only quantitative studies were included, but no restrictions were placed on specific study designs. This decision was made to comprehensively capture the breadth of available evidence on nutrition and physical activity interventions aimed at preventing or managing GDM. Limiting inclusion to a specific study design may exclude relevant research. Findings were interpreted within the context of study design, and greater emphasis was placed on higher-level evidence (e.g., randomized controlled trials (RCTs)). The population of interest included pregnant individuals with GDM and those at risk of developing GDM. Studies were included if they evaluated a nutrition lifestyle intervention, a physical activity lifestyle intervention, or a combined nutrition and physical activity lifestyle intervention that aimed to improve pregnancy outcomes. Eligible studies were required to assess GDM-related outcomes, including GDM incidence, insulin therapy rates, and/or blood glucose (BG) measures, reported as primary or secondary outcomes.

Studies were excluded if the intervention was not a nutrition or physical activity intervention (e.g., pharmacological interventions, self-glucose monitoring interventions). Interventions that focused only on dietary supplementation (e.g., Vitamin D, fiber, DHA) were also excluded, as the primary aim of this review was to evaluate lifestyle modification programs designed to promote sustained behaviour change. Although the potential benefit of Omega-3 intake was considered, only one study assessing DHA supplementation was identified, and it was excluded to maintain methodological consistency, as other supplement-only interventions were not included. Additionally, review articles, commentaries, secondary analyses, protocols without published outcomes, interventions with no GDM-related outcomes, articles with no English translation available, abstract-only publications, publications with no access to full articles, retracted articles, and articles with published error notices were excluded.

## 3. Results

### 3.1. Study Retrieval and Screening

**Figure 1:**
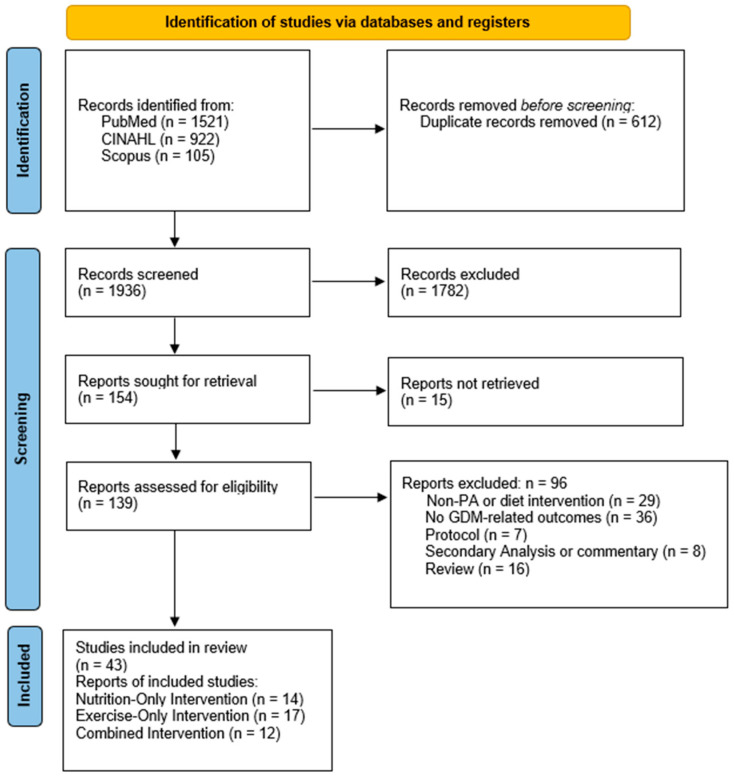
PRISMA diagram of article retrieval and screening.

A total of 2548 articles were identified during the initial database search. After excluding duplicates (n = 612), 1936 articles were screened by title and abstract to determine eligibility, and 154 articles remained after the first screening process. The remaining articles then underwent full-text analysis to determine eligibility. Forty-three articles met the inclusion criteria and were identified and included in the analysis. The screening and retrieval process was conducted by one reviewer; however, study selection criteria were predefined and applied consistently to enhance transparency. The comprehensive article screening process is displayed in Figure 1.

### 3.2. Nutrition-Only Interventions

Fourteen RCTs evaluated nutrition-only interventions for GDM prevention or management. Of those, nine were GDM management interventions [30,31,32,33,34,35,36,37,38], and five were GDM prevention interventions [39,40,41,42,43].

The included studies were conducted across a range of countries. Three studies were conducted in China [30,31,37], three in Spain [34,39,42], three in Iran [38,41,43], two in Australia [32,33], and one each in Norway [40], Brazil [34], and the United Kingdom [36].

GDM incidence was evaluated in three studies [39,41,43]. Two studies evaluating Mediterranean-style dietary patterns enriched with daily extra virgin olive oil and pistachio intake demonstrated statistically significant reductions in GDM incidence in the intervention groups compared with the controls [39,42]. One study, which evaluated a 0% trans-fat diet, reported no significant difference in GDM incidence between groups. However, the onset of GDM in the intervention group was significantly later than that in the control group (week 30 vs. week 24).

Insulin outcomes were assessed in seven studies [32,33,34,35,36,39,42]. Four studies reported a significantly lower number of patients requiring insulin in intervention groups, including a Mediterranean dietary intervention [39], an LGI intervention [33], a DASH diet intervention [38], and a restricted energy diet [36]. In the LGI trial, participants in the HGI group who started insulin therapy (n = 19) were transitioned to the LGI diet, after which nine no longer met criteria for insulin use [33]. The remaining interventions did not demonstrate any significant differences in insulin requirements between groups [32,34,35,42].

BGC outcomes were assessed in ten studies [30,31,32,35,36,37,38,39,40,41]. Trials implementing a Mediterranean diet [39], an LGI diet [31] intervention, and a DASH diet [38], as well as interventions incorporating digital follow-up and behavioural reinforcement through messaging platforms such as WeChat or WhatsApp [30,35,37], demonstrated significantly lower BGC in the intervention groups compared with the control groups. The remaining four studies did not report any significant differences in BGC outcomes between groups [32,36,40,41].

None of the nutrition-only trials incorporated culturally or religiously sensitive interventions.

Overall, interventions emphasizing LGI, Mediterranean, and DASH dietary patterns showed the most consistent improvements in glycemic outcomes and GDM incidence. Insulin-related outcomes were generally inconsistent throughout the interventions. No studies incorporated religiously or culturally sensitive interventions.

A comprehensive summary of nutrition-only interventions and outcomes is provided in Table 1.

### 3.3. Exercise-Only Interventions

One case–control [44], fifteen RCTs [45,46,47,48,49,50,51,52,53,54,55,56,57,58,59], and one quasi-experimental design [60] assessed the impact of exercise-only interventions. One study used a randomized design to compare two different exercise interventions but did not include a control group [50]. Of these, eleven studies were GDM management interventions [44,45,46,47,48,49,50,51,52,53,54], while six were GDM prevention interventions [55,56,57,58,59,60].

The studies were conducted in multiple countries, with five conducted in China [47,48,49,50,53], three in Canada [44,46,60], two in Spain [58,59], and one each in Turkey [45], Australia [46], Egypt [55], the Netherlands [56], Poland [51], Norway [56], and Switzerland [52].

GDM incidence was evaluated in four studies [57,58,59]. Two studies that incorporated structured and supervised online [58] and on-site [59] group exercise programs three times/week reported significantly lower GDM incidence in the intervention groups compared to the control groups. The remaining two studies, which incorporated a supervised exercise class once/week [57] and a prescribed, personalized home exercise program [60], reported no significant difference in GDM incidence.

Insulin outcomes were assessed in seven studies [44,48,50,52,53,54,56]. Of these, three interventions that incorporated supervised, on-site exercise programs delivered three times/week reported a significantly lower insulin usage rate in the intervention groups compared to the control groups [44,48,53]. One study, which evaluated a non-supervised resistance exercise program, did not report a difference in insulin use; however, participants in the intervention group had a later start to insulin as well as a lower dosage [54]. Three studies, all of which incorporated supervised exercise programs delivered one to three times per week, reported no significant differences in insulin outcomes between groups [50,52,56].

BGC outcomes were assessed in thirteen studies [44,45,46,47,48,49,50,51,53,54,55,56,59]. Of these, supervised and structured exercise programs delivered at least three times per week [44,45,48,53,55,59], along with exercise education and individualized programs that incorporated motivational interviewing and face-to-face follow-up sessions [47,49], were reported to have significantly lower BGC at the end of the intervention compared to control programs. Only one non-supervised exercise program intervention reported significantly decreased BGC in the intervention group compared to the control group [51]. One study that compared a supervised resistance training program to a supervised aerobics program, both delivered three times per week, did not report significant differences in BGC outcomes between groups. However, both groups demonstrated significantly lower BGC outcomes when pre- and post-values were assessed [50]. Three studies, which included a post-meal walking intervention compared to 30 min of continuous walking [46], a walking program of 5000 steps/day [56], and a supervised structured exercise program delivered twice per week [51], reported no significant BGC outcomes between groups.

None of the exercise-only trials incorporated culturally or religiously sensitive interventions.

Overall, exercise-only interventions implemented as structured, supervised programs of low–moderate intensity and performed three times per week were the most effective at improving glycemic and insulin-related outcomes, as well as preventing GDM incidence. No studies incorporated religiously or culturally sensitive interventions.

A comprehensive summary of exercise-only interventions and outcomes is provided in Table 2.

### 3.4. Combined Lifestyle Interventions

Twelve RCTs evaluated combined exercise and nutrition lifestyle interventions. Of these, five were GDM management interventions [61,62,63,64,65], and seven were GDM prevention interventions [66,67,68,69,70,71,72]. Of the included studies, five were conducted in China [61,64,65,68,70], three in Iran [62,69,71], and one each in the United States [66], Australia [67], India [63], and Finland [72].

GDM incidence was evaluated in seven studies [66,67,68,69,70,71,72]. Three studies implementing psychologically informed counselling interventions targeting diet and exercise reported significantly lower GDM incidence in intervention groups compared with control groups [67,68,71]. Four studies that included educational sessions along with a web-module [66] or reminder messages [69], as well as regular counselling sessions [70,72], did not report any significant difference in GDM incidence between groups.

BGC outcomes were assessed in seven studies, all of which reported significantly decreased and more controlled BGC outcomes in the intervention groups compared to the control groups [61,62,63,64,65,71,72]. No studies reported insulin outcomes.

One study conducted in Iran incorporated a religiously sensitive component within the intervention [62]. This study evaluated a mobile-assisted education program targeting women with GDM that included modules on nutrition, physical activity, and health-promoting lifestyle behaviours. Notably, the application also integrated a spiritual growth component as part of stress management, recognizing the religious context of the target population. Health-Promoting Lifestyle Profile scores were assessed as the primary outcome, and BGC was assessed as the secondary outcome. Both outcomes improved significantly in the intervention group compared with the control group following the intervention. Overall, combined lifestyle interventions that were focused on psychologically informed counselling were most effective in reducing GDM incidence, and all were successful in improving glycemic outcomes. No studies reported insulin-related outcomes, and only one study incorporated a religiously sensitive intervention.

A comprehensive summary of combined interventions and outcomes is provided in Table 3.

## 4. Discussion

### 4.1. Summary of Results

Across all intervention categories, combined lifestyle interventions appeared to be the most effective in improving glycemic control. This finding is consistent with the Cochrane review conducted by Griffith et al., which reported that combined dietary and exercise interventions may offer a beneficial effect in reducing the risk of GDM [25]. The Mediterranean diet appeared to be the most effective dietary intervention for reducing GDM incidence, while the Mediterranean, DASH and LGI diets were effective in managing GDM among diagnosed women. Dietary interventions that utilized digital tools for follow-up or for sending reminder messages were also effective in managing GDM. In the exercise category, supervised prenatal exercise programs of low to moderate intensity, lasting 45–60 min per session and delivered at least three times per week, along with interventions that involved motivation via behavioural theories, were consistently effective in either preventing or managing GDM. No clear differences were observed between aerobic, resistance, or combined aerobic and resistance programs with respect to GDM incidence, BGC, or insulin outcomes. These results are supported by the findings of other reviews. For example, Zhang et al. reported that DASH and LGI dietary patterns were effective in improving glycemic control among women with GDM [72], while Martinez-Vizcaino et al. concluded that supervised prenatal exercise interventions with low–moderate intensity significantly reduced the risk of GDM [73].

However, it is important to note that most of the existing literature examined GDM prevention and management interventions in isolation. For instance, several systematic reviews have focused exclusively on the role of lifestyle interventions in reducing the risk of developing GDM, while others have evaluated their effectiveness in managing glycemic outcomes among women already diagnosed with GDM [25,73,74]. This separation limits the ability to compare intervention effectiveness across the full continuum of care. In contrast, the present narrative review integrates both prevention and management studies, allowing for a comprehensive evaluation of how dietary, exercise, and combined lifestyle interventions perform across the different stages of GDM. This approach provides additional insight into the consistency of intervention effects and highlights the potential value of combined lifestyle strategies across both contexts. While many studies reported statistically significant improvements in glycemic outcomes, the clinical significance of these changes was not consistently reported, limiting the interpretation of their practical impact.

### 4.2. Adherence

Quantitative adherence reporting across all three intervention categories was heterogeneous, both in terms of whether it was reported and how it was measured. Across the included trials, nineteen reported adherence percentages [31,32,40,42,44,45,46,47,48,50,51,52,56,57,58,59,61,66,70]. Adherence was more commonly reported in exercise-only interventions compared to nutrition-only or combined interventions, likely because exercise participation can be objectively monitored through supervised sessions or wearable devices such as pedometers. In contrast, dietary intervention relied predominantly on self-reported measures, including food records, dietary recalls, and questionnaires, which are inherently more subjective and more difficult to quantify. Among the studies with reported adherence, it was found that when adherence exceeded 80%, improvements in at least one of the assessed outcomes [BGC, insulin, GDM incidence] were consistently observed [31,42,44,45,47,48,50,58,59,61].

### 4.3. Gestational Weight Gain (GWG)

Although pre-pregnancy overweight, obesity, and excessive GWG are well-established risk factors for GDM, the role of weight management within the included studies was less defined. Of the included studies, a few reported GWG as a primary outcome [33,48,63,64,65], while the rest either did not report GWG or reported it as a secondary outcome. GWG was rarely analysed in relation to glycemic measures in the included studies; therefore, the independent effect of GWG on GDM incidence or glycemic outcomes cannot be determined from the findings of this narrative review.

### 4.4. Heterogeneity Across Studies

There was substantial heterogeneity across studies in terms of diagnostic criteria, intervention design, outcome measurement, and reporting practices, which limits direct comparison and synthesis of findings. Variability was evident in the diagnostic criteria used to define GDM. Although most of the studies used IADPSG/WHO criteria, many studies applied alternative standards, including CDA, ADA, and Carpenter–Coustan criteria. These differing diagnostic thresholds may have influenced the reporting and comparability of GDM incidence across the studies. Intervention intensity and structure also differed considerably, ranging from brief educational counselling sessions to highly supervised and structured programs delivered several times a week. Intervention duration also varied, with some lasting as little as five days while others extended over several weeks or throughout pregnancy.

Outcome reporting was similarly inconsistent. The studies assessed different glycemic parameters (FBG, HbA1c, iAUC, PBG, etc.), and adherence measurements varied across interventions. The reporting of effect sizes and percentage changes were also inconsistent, with many studies failing to provide these metrics, thereby limiting the ability to evaluate the magnitude and clinical relevance of observed effects. Furthermore, baseline characteristics were not uniformly reported; some studies provided detailed participant profiles, whereas others reported only limited baseline data, which may have influenced the interpretation of intervention effects and between-study comparisons.

### 4.5. Literature Gaps and Future Research Implications

Only one study incorporated a religiously sensitive component. This limited evidence base represents an important finding of the current review and highlights a significant gap in the literature, rather than a limitation of the narration.

Research conducted in Canada by Read et al. demonstrated that non-Caucasian women with GDM had more non-modifiable risk factors, including lower socioeconomic status and education levels, along with a stronger family history of diabetes. On the other hand, Caucasian women were more likely to present modifiable risk factors such as smoking or alcohol use prior to pregnancy [75]. Similarly, Banerjee et al. reported health behaviour changes after a GDM diagnosis among ethnic women living in Canada. Subgroup analysis showed that readiness to change differed by ethnicity, where some groups were more inclined to increase physical activity, whereas others were more likely to modify dietary behaviours, such as portion size reduction [76]. These findings suggest that risk profiles vary across ethnic groups, and interventions should be sensitive to the culture or religion of these groups. This approach is supported by a scoping review conducted by Sympath et al., which demonstrated that culturally appropriate diabetes care was associated with improved outcomes in Indigenous communities [77].

Another important gap identified in the current review is the lack of insulin-related outcomes reported in the combined lifestyle interventions. Although combined diet and exercise strategies demonstrated strong evidence for improving glycemic control, none of the studies examined insulin initiation, dosage requirements, or progression to insulin therapy. This limits the ability to determine whether improved glycemic control translated into clinically meaningful reductions in pharmacological treatment. Future research should incorporate insulin outcomes when evaluating combined lifestyle interventions to better assess their full clinical impact.

### 4.6. Strengths and Limitations

This narrative review comprehensively examined dietary, exercise, and combined lifestyle interventions for both the prevention and management of GDM. It also uniquely incorporated an evaluation of culturally and religiously sensitive interventions, which other reviews on this topic do not cover. In doing so, the current manuscript provided currently relevant insights, as well as identified important gaps in the literature.

In addition to the strengths, it is also important to note the limitations. As this is a narrative review, a formal risk of bias assessment was not conducted, which limits the ability to evaluate the strength of the evidence. Additionally, inconsistent reporting of effect sizes and outcome measures across studies limits the ability to assess the magnitude and clinical relevance of observed effects.

## 5. Conclusions

In conclusion, this review highlights the important role of lifestyle in both the prevention and management of GDM, with evidence suggesting that combined dietary and exercise approaches appear to be the most consistently associated with improvements in glycemic outcomes. Specific dietary patterns such as the Mediterranean, DASH, and LGI diets alongside regular low–moderate-intensity prenatal exercise demonstrate consistent benefits. However, the limited reporting of insulin-related outcomes in combined lifestyle interventions and the near absence of culturally or religiously sensitive interventions represent critical gaps in the literature. Addressing these gaps is necessary to expand the literature and improve the evidence base surrounding GDM prevention and management.

## Figures and Tables

**Table 1:** Nutrition intervention results.

Author, Year (Country)	Objective + GDM Diagnosis Method	Sample Size	Estimated Sample Size	Outcome Measures	Intervention Design and Description	Adherence + Duration	Baseline Characteristics
Yuan et al., 2020 (China) [30]	To explore the effects of 12 h nutrition care in patients with GDM with the aim to establish a more rational and effective prenatal care model. Criteria: IADPSG/WHO.	Lost to follow-up: not reported Analysed: n = 312 nIG = 158, nCG = 154	Not reported	Primary and secondary not mentioned. Gestational weight gain * (GWG *) Fasting Blood Glucose (FBG) 2hPostprandial blood glucose * (2hPBG *) (percent change not provided) Gestational HTN * Preterm labor * Neonatal BW * Neonatal hypoglycemia	RCT IG: Patients accompanied for 12 h in-hospital to demonstrate a full day of the GDM diet. WeChat group created for follow-up and questions after discharge. CG: Standard care.	Adherence: Not reported Duration: From GDM diagnosis to delivery	Age BGC Education BMI
Hu et al., 2014 (China) [31]	To investigate the influence of an LGI diet on PBG in women with GDM. Criteria: IADPSG/WHO.	Lost to follow-up: not mentioned Analysed: n = 140 nIG = 6 nCG = 74	Not reported	Primary: FBG Breakfast PBG * (−18.7 SD 14) Lunch PBG * (−20.3 SD 11.9) Dinner PBG * (−22.1 SD 13.4)	RCT. All subjects hospitalized for 5 days and provided with diet in-hospital. IG: LGI diet that replaced white rice in lunch and dinner with LGI alternative. CG: Standard diet with white rice.	Adherence: 100% Duration: 5 days	Age Gestational age Pre-pregnancy weight and BMI Parity Family history of diabetes BGC
Assaf-Balut et al., 2017 (Spain) [39]	To assess the effect of an intervention based on MedDiet reinforced with abundant extra virgin olive oil (EVOO) and nuts in the for of pistachios on the incidence of GDM at 24–28 gestational weeks (GW). Criteria: IADPSG/WHO.	Lost to follow-up: nCG = 60 nIG = 66 (reasons: miscarriage, no GDM screening information, change of hospital) Analysed: n = 874 nIG = 434 nCG = 440)	Assuming a decrease in FBG of −7 mg/dL 3 months post-MedDiet, the authors estimated 315 women were needed per group to provide a statistical power of 80% (two-tailed, α error of 0.05) and to detect a relative risk reduction of at least 30%, with a projected incidence of 35% GDM in the control group.	Primary: GDM incidence * (RR:0.75; 95% CI: 0.57–0.98) Secondary: Insulin prescription * (RR:0.43; 95% CI: 0.24–0.78) FBG (percent change not provide) PBG * HbA1c * GWG * Gestational HTN C-section Preterm delivery * Perineal trauma * SGA * LGA * NICU admission MEDAS score *	Prospective RCT IG: Standard MedDiet with specific instructions to consume 40 mL EVOO and handful of pistachios daily. CG: Standard care.	Adherence: Not reported Duration: Throughout pregnancy	Age Race Family history of T2DM GDM history Miscarriage Education status Employment Number of pregnancies Smoking history Gestational age Pre-pregnancy BW BP FBG TSH MEDAS score Physical activity
Louie et al., 2011 (Australia) [32]	To investigate the effect of an LGI diet vs. high-fiber diet on pregnancy outcomes, neonatal outcomes, and maternal metabolic profile in GDM. Criteria: IADPSG/WHO.	Lost to follow-up: nIG = 4 nCG = 3 (reasons: withdrawn) Analysed: n = 92 nIG = 47, nCG = 45	Based on previous data, the study was designed to provide 80% statistical power to detect an ∼260 g difference in birth weight, with 60 subjects in each group.	Primary: neonatal BW Secondary: Other pregnancy outcomes Insulin HOMA-IR HbA1c Fructosamine	RCT. GI table provided to both groups. IG: Instructed to consume LGI foods only. CG: Instructed to consume mod-high GI foods, with high fiber.	Adherence: 88% Duration: From GDM diagnosis to delivery	Age Pre-pregnancy BMI Ethnicity GW at diagnosis T2DM family history Nulliparous 75 g OGTT results BGL Insulin HOMA-IR HbA1c Fructosamine
Skarstad et al., 2024 (Norway) [40]	To examine the adherence to time-restricted eating (TRE) in pregnant individuals with at least one risk factor for developing GDM. Criteria: 2017 Norwegian Criteria.	Lost to follow-up: nIG = 1 nCG = 1 (reason: withdrew consent) Analysed: n = 32 nIG = 15 nCG = 17	A formal power analysis was not performed. Generally, a sample size between 24 and 50 is recommended to estimate the effect size and standard deviation in feasibility studies.	Primary: Adherence Secondary: PA behaviour Total Energy Intake (TEI) iAUC	RCT. IG: TRE program with a max of 10 h daily eating window. CG: No TRE.	Adherence: 67% Duration: 5 weeks	Age Gestational week Parity Height Weight BMI Muscle mass Fat mass Fat percentage Visceral fat Blood pressure FBG HbA1c HOMA-IR HDL LDL Triglycerides
Moses et al., 2009 (Australia) [33]	To determine whether prescribing an LGI diet for women with GDM could reduce the number of women requiring insulin without compromise of pregnancy outcomes. Criteria: ADIPS.	Lost to follow-up: none Analysed: n = 63 nIG = 31 nCG = 3 n = 19 from CG transferred to LGI after insulin prescription	Not reported	Primary: Insulin requirements * (effect size not provided) Secondary: GWG Labor induction LGA SGA	RCT. IG: Instructed to consume LGI foods only. CG: Instructed to consume a high-fiber and low-sugar diet with mod-high GI foods.	Adherence: Not reported Duration: From GDM diagnosis to delivery	Age Weight and BMI at enrolment Parity OGTT results Gestational age at enrolment
Moreno-castilla et al., 2013 (Spain) [34]	To test the hypothesis that a low-CHO diet for the treatment of GDM would lead to a lower rate of insulin treatment with similar pregnancy outcomes compared with a control diet. Criteria: NDDA.	Lost to follow-up: nIG = 15 nCG = 6 (reasons: withdrew) Final analysed: n = 150 nIG = 75 nCG = 75	An 80% statistical power (95% bilateral confidence to find a 22% minimum difference on risk of insulinization (expected rate of insulin-treated women in the CG was 45%), with anticipated 10% loss. A total of 152 participants required.	Primary: Insulin prescription Secondary: Gestational age at delivery GWG * Ketonuria Gestational HTN C-section SGA LGA Macrosomia Neonatal hypoglycemia	RCT. IG: 20% protein, 40% fat, and 40% carb from diagnosis to delivery. CG: 20% protein, 25% fat, and 55% carb.	Adherence: Not reported Duration: From GDM diagnosis to delivery	Age Pre-pregnancy BMI Gestational age OGTT results Non-Caucasian Nulliparous Never smoking Pre-pregnancy HTN
Sharifat et al., 2024 (Iran) [41]	To evaluate the effect of GI training based on the health-belief model (HBM) on metabolic profile and health-related quality of life among pregnant mothers at risk of GDM referring to primary health centers. Criteria: Not reported.	Lost to follow-up: nIG = 3 nCG = 3 (reason: withdrawal, abortion) Analysed: n = 84 nIG = 42, nCG = 42	Considering a mean (SD) of 84.81 (8.16), a power of 90%, and 95% confidence, accounting for 30% dropout, a calculated sample size of 45 women was required in each group.	Primary: FBG OGTT results HBM scores Secondary: Dietary intake Physical activity GWG Fasting insulin * HOMA-IR HOMA-IS Health-related QOL hs-Crp * Total cholesterol * LDL HDL Triglycerides *	RCT. IG: Two face-to-face, group education sessions about GI and GL given to groups of 8–10 women at a time, based on HBM theory. CG: Standard care.	Adherence: Not reported Duration: From GDM diagnosis to delivery	Age Pre-BMI GWG METs/week * Income status Living with old person Number of children * Mother job status Pregnancy intention Mother education Spouse education Folic acid supplement Iron supplement
Valença et al., 2024 (Brazil) [35]	To compare the effectiveness of outpatient nutritional guidance supplemented by digital media with exclusively standard outpatient care nutritional guidance in pregnant women with GDM. Criteria: IADPSG/WHO,	Lost to follow-up: n = 5 (reason not mentioned) Analysed: n = 81 nIG = 34 nCG = 47	Statistical significance of 5% was considered, with an 80% test power and an effect size of 0.65. A minimum of 78 women required.	Primary and secondary not specified Insulin therapy Glycemic control * (percent change not provided) Dietary intake	RCT. IG: Six daily reminders sent via WhatsApp in addition to standard outpatient care. CG: Standard outpatient care.	Adherence: Not reported Duration: 4–8 weeks	Age Number of pregnancies BMI at inclusion Ethnicity Marital status No significant difference between groups
Martín-O’connor et al., 2025 (Spain) [42]	To evaluate whether a video intervention guided by a nutritionist could increase compliance to the Mediterranean diet and reduce the incidence of GDM and adverse maternal–neonatal outcomes. Criteria: IADPSG/WHO.	Lost to follow-up: nIG = 108 nCG = 9 (reasons: could not download video, lack of GDM screening) Final analysed: n = 1529 nIG = 715 nCG = 814.	Assuming a baseline prevalence of 25% in the CG, the authors aimed to detect a 5% absolute reduction in IG (from 25% to 20%). For a two-sided comparison of two proportions (α = 0.05, power = 80%), and a 5% drop-out rate, 662 women were required per group.	Primary: GDM incidence * (4.4% difference) Secondary: GWG Insulin therapy Gestational HTN * Preeclampsia Delivery mode Preterm birth incidence Neonatal hypoglycemia Neonatal BW MEDAS score * IPAQ score	RCT. IG: AN 8 min video explaining MedDiet (emphasis on EVOO and pistachio daily intake) and physical activity recommendations during pregnancy. Participants followed from enrolment to delivery. CG: Standard care.	Adherence: 100% Compliance defined by viewing video at least once Duration: From enrolment to delivery	Age Pre-pregnancy Weight Pre-pregnancy BMI sBP dBP FBG HbA1c Primiparous Prior GDM Prior miscarriage Never smoked Ethnicity * Educational status Occupation MEDAS score
Kusinski et al., 2025 (UK) [36]	Not explicitly mentioned; to assess if a reduced-energy diet of 1200 calories/day prevents EGWG in women with GDM. Criteria: NICE.	Lost to follow-up: nIG = 16 nCG = 21 (reasons not mentioned) Analysed: n = 388 nIG = 198 nCG = 190	The probability of finding the original effect size was calculated to be 0.72 if 380 women were recruited and 0.85 for both outcomes if 500 women were recruited. The data safety monitoring board therefore considered that the trial should not be stopped for futility after n = 250, but that 380 participants was sufficient to identify if significant differences were present.	Primary: GWG * Secondary: C-section Long-acting insulin therapy * (OR:0.36; 95% CI: 0.18–0.7) Short-acting insulin Metformin HbA1c BP NICU admission Estimated gestation at birth	RCT. IG: Delivered 1200 cal/day diet box from GDM to delivery. CG: Delivered 2000 cal/day diet box.	Adherence: Not reported Duration: From GDM diagnosis to delivery	Age BMI Ethnicity Primiparous GWG pre-enrolment Maternal education GDM history Smoking Physical activity Habitual TEI BMR BP Gestational age at diagnosis OGTT results Metformin use Short-acting insulin use Long-acting insulin use
Alamolhoda et al., 2019 (Iran) [43]	To examine the effect of low trans-fatty acid (TFA) intakes during pregnancy on GDM. Criteria: Carpenter–Coustan.	Lost to follow-up: n = 800 nIG = 62 nCG = 50 Analysed: n =800 nIG = 393 nCG = 407	Not reported	Primary: GDM incidence Onset of GDM incidence * (effect size not provided)	RCT IG: Individualized: 0% dairy products, olive oil for cooking, less than 1% TFA, and prohibited from eating deep-fried and fast food. CG: Individualized diet: 1.5% dairy, liquid oil, and advised to avoid deep-fried and fast food.	Adherence: Not reported Duration: From enrolment to delivery	Age Pre-pregnancy BMI Blood pressure Housewife occupation High school education Nulli porous Para Abortion
Pan et al., 2025 (China) [37]	To investigate the effects of a Cognitive Behaviour Theory (CBT)-based digital dietary intervention on glycemic control and pregnancy outcomes in patients with GDM. Criteria: IADPSG/WHO.	Lost to follow-up: nIG = 15 nCG = 14 (reasons not mentioned) Analysed: n = 171 nIG = 85 nCG = 86	To detect a difference of 65% GQR in the control group and an anticipated mean difference of 20% between the two groups, a 5% significance level, with a power of 80%, and 20% drop-out rate, a min. of 100 participants per group was required.	Primary: Glycemic Qualification Rate (GQR) * (effect size not provided) Secondary: FBG, Breakfast PBG **Lunch PBG** * (−0.2 SD 0.7) Dinner PBG * (−0.2 SD 0.8) General Self-Efficacy Scores * Macrosomia *	RCT. IG: A 12-week, structured dietary, CBT-based digital intervention delivered via WeChat that focused on appropriate food selection and meal sequencing. CG: Standard care.	Adherence: Not reported Duration: 12 weeks	Age Gestational age Height Weight OGTT—2 h FBG HbA1c Immediate family T2DM history GQR
Asemi et al., 2013 (Iran) [38]	To investigate the effects of the DASH diet on insulin resistance, serum high-sensitivity C-reactive protein (hs-CRP) and biomarkers of oxidative stress among pregnant women with GDM. Criteria: ADA.	Lost to follow-up: nIG = 3 nCG = 3 (reasons: bed rest, preeclampsia, insulin therapy) Analysed: n = 32 nIG = 16 nCG = 16	Not reported	Primary and secondary not mentioned. FBG * (percent change not provided) Serum insulin * HOMA-IR * hs-CRP Mode of delivery * Neonatal BW *	RCT IG: DASH diet. CG: Standard diet.	Adherence: Not reported Duration: From GDM diagnosis to delivery	Age Height Pre-pregnancy weight and BMI OGTT results

* = Statistically significant difference between-group comparisons; IG = intervention group, CG = control group.

**Table 2:** Exercise intervention results.

Author, Year (Country)	Objective	Sample Size	Estimated Sample Size	Outcome Measures	Intervention Design Description	Adherence + Duration	Baseline Characteristics
Davenport et al., 2008 (Canada) [44]	To investigate the effect of a structured low-intensity walking protocol (at 30% of heart rate reserve beginning at 25 min/session and building to 40 min) on capillary glucose concentrations and insulin requirements in women with GDM. Criteria: CDA.	Lost to follow-up: not reported Analysed: n = 30 nIG = 10 nCG = 20	Not reported	Primary: Subjects requiring insulin Frequency of insulin injections * (effect size not provided) Continuous capillary glucose * Secondary: GWG Macrosomia C-section	Case–control. CG: Bi-weekly, RD counselling to achieve nutritional goals of 2000 cals/day with 200 g/d carbs spread over three meals and three snacks. IG: Bi-weekly RD counselling and a supervised walking intervention.	Adherence: 100% Duration: 6 weeks	Participants recruited were matched to controls similar in pre-pregnancy BMI, age, and insulin use.
Menek and Kaya, 2024 (Turkey) [45]	To compare the efficacy of a self-directed home exercise program, standard care alone and a supervised home exercise program in pregnant women with gestational diabetes on blood glucose, quality of life and pregnancy outcomes. Criteria: ADA.	Lost to follow-up: nIG1 = 1 nIG2 = 1 nCG = 2 (reasons: poor exercise compliance, orthopedic problems) Analysed: n = 45 nIG1 = 15 nIG2 = 15 nCG = 15	Estimated to be 42 with 80% power (α = 0.05, β = 0.20) and effect size (F = 0.25) using the G power sample size calculator (G Power, v.3.0.10)	Primary: FBG * PBG * (effect sizes not provided) **WHOQOL-BREF scores** * Secondary: Neonatal BW Mode of delivery	RCT. Group 1: Supervised home exercise program: three low–mod intensity aerobic and resistance exercise sessions/week. Group 2: Home exercise program: patients received home exercise brochure with no further instructions. CG: Standard prenatal care.	Adherence: Group 1: 15/16 = 94% Group 2: not reported Duration: 8 weeks	Age Number of pregnancies GWG OGTT—results QOL results
Christie et al., 2024 (Australia) [46]	To determine whether advice to perform post-meal walking could be an effective and feasible alternate to standard care continuous walking for the management of gestational diabetes (GDM). Criteria: WHO.	Lost to follow-up: nIG = 8 nCG = 6 (reasons: personal reasons and skin irritation) Analysed: n = 32 nIG = 12 nCG = 14	Forty women needed to achieve 80% power to detect a clinically relevant 20% difference in postprandial hyperglycemia, assuming a ∼25% drop-out rate in the population.	Primary: iAUC METs/week **Sedentary time** * (66 mi/d SD 137; 95% CI: 13.16–120.24) Secondary: Mode of delivery Neonatal outcomes	RCT. IG: Advised to distribute 30 min of exercise/day into 10 min slots within 60 min after each meal. CG: Advised to walk 30 min/day.	Adherence: IG = 17% CG = 0% Duration: 5 days	Age Height Weight **Pre-pregnancy BMI** Parity Godin leisure score
Liu et al., 2025 (China) [47]	To analyse the application of a shared decision-making (SDM)-based exercise management program in patients with gestational diabetes mellitus (GDM) and its impact on blood glucose control. Criteria: Not reported.	Lost to follow-up: not reported Analysed: n = 88 nIG = 44 nCG = 44	Not reported.	Primary and secondary not mentioned. Compliance with medical advice * DSQL score * (SDSCA score * FBG * HbA1c * PBG * (difference not reported) Maternal and neonatal outcomes	Retrospective RCT. CG: Conventional exercise program that included health education and exercise recommendations. IG: Individualized exercise management plan based on SDM and motivational interviewing (MI) for increasing adherence.	Adherence: CG = 61% IG = 81% Duration: From GDM diagnosis to delivery	Age Gestational age Pre-pregnancy BMI Number of pregnancies Primiparity Family history of diabetes GDM history Educational level
Xie et al., 2021 (China) [48]	To investigate the effects of structured moderate-intensity aerobic exercise on blood glucose, insulin, and pregnancy outcomes in patients with gestational diabetes mellitus (GDM). Criteria: WHO and IADPSG.	Lost to follow-up: nIG = 8 nCG = 4 (reasons: hospital transfer, unknown, poor compliance) Analysed: n = 89 nIG = 43 nCG = 46	In the study, a significance level of α = 0.05 and a test efficiency of 1 − β = 0.8 were set for the bilateral test.	Primary: FBG * 2hPBG * Insulin usage * Insulin utilization * (effect size not reported) Adverse events Macrosomia C-section Preterm birth Agpar scores	RCT. IG: A total of 60 min of supervised aerobic group exercise preformed 3×/week on-site. CG: Standard care.	Adherence: IG: 84% Duration: 6 weeks	Age Education Parity C-section history Family history of diabetes Current gestational age Height Weight and BMI before pregnancy Weight at GDM diagnosis OGTT results BGC pre-intervention
He et al., 2024 (China) [49]	To evaluate the effects of multidimensional quantitative exercise management on self-efficacy, blood glucose control, and delivery outcomes in pregnant women with gestational diabetes mellitus (GDM). Criteria: Diagnosis and Treatment Guidelines for Gestational Diabetes Mellitus (2014).	Lost to follow-up: not reported Analysed: n = 150 nIG = 75 nCG = 75	*P*_2_ = 0.226. In the study, a 25% improvement in the intervention group was expected, i.e., P_1_ = 0.467. Considering a 20% dropout rate, the calculated sample size was 75.	Primary and secondary not mentioned. FBG * 2hPBG * HbA1c * (differences not reported) P-ESES scores GWG * Gestational infections VAS * Agpar scores *	RCT IG: Multidimensional healthcare team, development of personalized exercise prescriptions and regular face-to-face or remote exercise guidance. CG: Standard care.	Adherence: Not reported Duration: 6 weeks	P-ESES FBG 2hPBG
Xie et al., 2022 (China) [50]	To investigate the effect of resistance exercise versus aerobic exercise on blood glucose level, insulin utilization rate, and pregnancy outcome in patients with GDM. Criteria: Not reported.	n = 100 Lost to follow-up: nIG = 6, nCG = 8 (reasons: poor exercise compliance, out-of-hospital delivery) Analysed: n = 86 nIG = 43 nCG = 43	The significance level α = 0.05 and the test efficiency 1 − β = 0.8 were set for bilateral tests with the assumption of 20% lost to follow-up, requiring 48 per group.	Primary and secondary not mentioned. FBG PBG Insulin utilization Patient compliance * Adverse events Insulin use Pregnancy outcome	RCT IG: Six-week, supervised, 50–60 min resistance exercise program three times/week. CG: Supervised, 50–60 min aerobic exercise program performed three x/week.	Adherence: IG = 42/43 = 98% CG = 35/43= 81% Duration: 6 weeks	Age Education Parity number Family history of diabetes C-section history Current gestational age Height Pre-pregnancy weight and BMI Gestational age at GDM diagnosis OGTT results Pre-intervention BGC
Embaby et al., 2016 (Egypt) [55]	To assess the effect of aerobic exercises on insulin sensitivity and fasting plasma glucose level in pregnant women with risk for gestational diabetes mellitus. Criteria: Not reported.	Lost to follow-up: Not reported Final analysed: n = 40 nIG = 20 nCG = 20	Not reported.	Primary: FBG * (percent difference not reported) Fasting insulin *	RCT IG: Structured and supervised 45 min aerobic program, three x/week CG: Standard care.	Adherence: Not reported Duration: 12 weeks	BMI Age FBG Fasting insulin
Oostdam et al., 2012 (Netherlands) [56]	To evaluate the effectiveness of an exercise program for pregnant women with overweight or obesity and at risk for gestational diabetes mellitus (GDM). GDM: Not reported.	Lost to follow-up: T1: n = 8 T2: n = 22 Analysed: T1: nIG = 48 nCG = 51 T2: nIG = 40 nCG = 45	Adequate power (>0.80) and a 5% significance level would be achieved with 80 pregnant women in both groups. The power calculation allowed for a 20% drop-out rate.	Primary and secondary not specified. Insulin therapy FBG HbA1c Fasting insulin GWG Daily PA Neonatal outcomes	RCT. IG: Supervised, 60 min mixed aerobic and strength session, two x/week CG: Standard care.	Adherence: 16.3% attended at least half of the training sessions Duration: From enrolment to delivery	Age BMI Parity Race/ethnicity Educational level Employment status
Adamczak et al., 2024 (Poland) [51]	To analyse the impact of the use of pedometers to supervise physical activity on maternal health and the obstetric outcomes of pregnant women with obesity and early gestational diabetes. Criteria: PDA.	Lost to follow-up: nIG = 9 (reason: poor compliance) nCG = 0 Analysed: n = 115 nIG = 53, nCG = 62	To achieve 80% power at a two-sided 5% significance level, and to detect between-group differences of 4 kg in GWG with 7 kg SD, 60 participants required per group.	Primary: GWG HbA1c Maternal anthropometrics Secondary: Neonatal BW Gestational age SGA LGA	RCT. IG: Advised to take 5000 steps/day and provided with pedometers. CG: Advised to take 5000 steps/day with no pedometers provided.	Adherence: IG = 24/53 = 45% Duration: From GDM diagnosis to delivery	Age * Gestational age Parity Miscarriage history * Pre-pregnancy weight and BMI Family history of obesity, diabetes, and HTN
Stafne et al., 2012 (Norway) [57]	To assess whether exercise during pregnancy can prevent gestational diabetes and improve insulin resistance. Criteria: WHO.	Lost to follow-up: nIG = 54 nCG = 99 (reasons: illness, moved, no reason, medical) Analysed: n = 702 nIG = 375 nCG = 327	Assumed a GDM risk difference of 5% between groups. Under these assumptions, a two-sample comparison with a 5% level of significance and power of 0.80 gave a study population of 381 patients in each group.	Primary: GDM incidence Insulin resistance * Secondary: GWG BMI Pregnancy complication Neonatal outcomes	RCT. IG: Standardized, 60 min, group exercise program once/week. Encouraged to follow a written, 45 min home exercise program two x/week. CG: Standard care.	Adherence: IG = 55% exercising 3 x/week CG = 10% exercising 3 x/week Duration: 12 weeks	Age BMI Parity GDM Gestational HTN Exercise three x/week Booking weight OGTT HOMA IR Blood pressure Insulin resistance *
Uria-Minguito et al., 2022 (Spain) [58]	To examine the effect of an online supervised exercise program during pregnancy on the prevention of GDM and on maternal and childbirth outcomes. Criteria: NDDA.	Lost to follow-up: nIG = 28 nCG = 29 (reasons: low adherence, change of hospital, medical issues, other) Analysed: n = 303 nIG = 102 nCG = 101	Following a two-sample comparison (χ^2^) with a 5% level of significance, statistical power of 0.90, and 25% lost to follow-up, 130 women were recruited.	Primary: GDM incidence * (OR: 0.255; 95% CI: 0.09–0.72) Secondary: GWG * Mode of delivery * Neonatal outcomes	RCT. IG: Structured and supervised, 5 min, online exercise program, 3x/week. Two sessions via zoom, and one session pre-recorded on YouTube. CG: Standard care.	Adherence: IG: 91/102 = 89% Duration: From enrolment to delivery	Age Height Weight BMI Parity Smoking during pregnancy Occupation Previous miscarriage
Barakat et al., 2012 (Spain) [59]	To study the influence of an exercise program performed by healthy pregnant women on maternal glucose tolerance. Criteria: Carpenter–Coustan.	Lost to follow-up: nIG = 10 nCG = 7 (reasons: medical and personal reasons) Analysed: n = 83 nIG = 40 nCG = 43	Not reported.	Primary: 50 g MGS *(difference not reported) GDM incidence * (Size effect not reported) GWG * Secondary: Mode of delivery Agpar score Neonate BW	RCT. IG: Structured, on-site, 35–45 min session preformed three x/week, with two land aerobic sessions and one aquatic session throughout pregnancy. CG: Standard care.	Adherence: IG: 85% Duration: From enrolment to delivery	Age BMI Smoking habits Alcohol intake Occupational activity Hours standing Maternal education * Parity Exercise habits pre-gestation
Boulvain et al., 2024 (Switzerland) [52]	To assess the effectiveness of an exercise intervention, in addition to standard care, in preventing or delaying insulin prescription in women with gestational diabetes mellitus (GDM). Criteria: Carpenter–Coustan or IADPSG.	Lost to follow up: nIG = 2 nCG = 0 (reasons: error of inclusion) Analysed: n = 107 nIG = 55 nCG = 52	To detect a clinically relevant reduction to 20% in the intervention group (number-needed-to-treat of five), the authors calculated a required sample size of 91 patients per group (α = 0.05, 80% power).	Primary: Insulin prescription Secondary: Insulin initiation Insulin dose Mode of delivery Neonatal birthweight and morbidity	RCT IG: Weekly, 30–45 min, aerobic and light resistance training exercise program in-hospital along with motivational interviewing to increase daily steps to 5000 from diagnosis to delivery. CG: Standard care.	Adherence: IG: 30% Duration: From GDM diagnosis to delivery	Age Nulliparous * women Smoker Ethnic group Weight before pregnancy and at randomization BMI * Height Type of GDM screening
Saidi et al., 2023 (Canada) [60]	To assess associations between an intervention including PA education by prenatal nurses and a PA prescription delivered by physicians on fetal and maternal outcomes. Criteria: CDA.	Lost to follow-up: nIG = 43 nCG = 0 (reasons: medical) Analysed: n = 816 nIG = 422 nCG = 394	A total of 369 per group would provide a power of 80% with a 5% alpha error probability of noting an increase in the proportion of women who will gain the recommended weight if it reaches 43% in the group exposed to an enhanced care offer.	Primary and secondary not mentioned. GDM incidence GHT incidence * GWG Induction of labor Mode of delivery Perineal tears Neonatal BW *	Quasi-experimental. IG: Educational materials, individualized GWG tracking, and personalized exercise prescription with ongoing monitoring and support. CG: Standard care.	Adherence: Not reported Duration: Throughout pregnancy	Age Height Pre-pregnancy weight BMI Marital status Education Ethnicity Gestational age Parity GDM history Gestational HTN history
Zhao et al., 2022 (China) [53]	To investigate the effects of a structured moderate-intensity resistance exercise program on blood glucose levels and other health-related indicators in patients with GDM. Criteria: ADA.	Lost to follow-up: nIG = 6 nCG = 4 (reasons: medical, one due to poor exercise compliance) Analysed: n = 96 nIG = 49 nCG = 46	A bilateral test was conducted, and a significance level of α = 0.05 and a test efficacy of 1 − β = 0.8 were set. Assuming a 20% loss to follow-up rate, a minimum of 48 was required/group.	Primary: FBG * PBG * (difference not reported) Secondary: Insulin prescription * Insulin dosage * (size effect not reported) Preterm delivery C-section Macrosomia GWG * Blood pressure * Adverse events	RCT. IG: Resistance exercise three times/week for 6 weeks at least. CG: Standard care.	Adherence: Not reported Duration: 6 weeks	Age Education parity C-section history Family history diabetes Gestational age Height Pre-pregnancy weight and BMI Weight at GDM diagnosis OGTT results
Brankston et al., 2004 (Canada) [54]	To examine the effects of circuit-type resistance training on the need for insulin in women with gestational diabetes mellitus. Criteria: CDA.	Lost to follow-up: nIG = 2 nCG = not reported (reasons: medical) analysed: n = 32 nIG = 16 nCG = 16	To calculate the sample size, it was estimated that the incidence of insulin use would need to be reduced to 25% to be considered clinically significant. For 0.8 power and an α-value of 0.05, 32 subjects would be required.	Primary: Insulin prescription Secondary: Insulin initiation time * Insulin dose * (effect size not provided) FBG * PBG * (difference not provided)	RCT IG: Four-week, non-supervised, resistance exercise program (first three sessions supervised). CG: Standard care in IG compared to CG.	Adherence: Not reported Duration: 4 weeks	Age Height Pre-pregnancy weight * Pre-pregnancy BMI Gestational age Weight up to diagnosis GWG GDM history

* = Statistically significant difference between-group comparisons; IG = intervention group; CG = control group.

**Table 3:** Combined nutrition and exercise intervention results.

Author, Year (Country)	Objective + GDM Diagnosis Method	Sample Size	Estimated Sample Size	Outcome Measures	Intervention Design and Description	Adherence + Duration	Baseline Characteristics
Jing and Liu et al., 2025 (China) [61]	To examine how an SFEE diet management intervention affected glycemic control, maternal outcomes, and dietary compliance in GDM. Criteria: Not reported.	Lost to follow-up: nIG = 2 nCG = 2 (medical reasons) Final analysed: n = 60 nIG = 30 nCG = 30	Alpha set at 0.05 and beta set at 0.10. Reference values from the literature were used for the standard deviation (7.95) and difference between means (7). The sample size was adjusted for loss to follow-up at 20% so that 64 subjects were ultimately enrolled.	Primary: FBG * (−0.5 mmol/L; 95% CI: −0.8 to −0.2) PBG * (−1.2 mmol/L; 95% CI: −1.7 to −0.7) HbA1c * (−0.03; 95% CI: −0.6 to −0.1) Secondary: Neonatal BW C-section * Macrosomia * Adherence * GDM knowledge * Perceived social support *	RCT IG: Self–family–environment–empowerment (SFEE) intervention via seven face-to-face sessions given bi-weekly for GDM management through diet and exercise post GDM diagnosis. CG: Standard care.	Adherence: IG = 85% CG = 60% Duration: 14 weeks	Age Physical fitness pre-pregnancy FBG 1hPBG 2hPBG Parity Family history of diabetes GDM history
Chang et al., 2023 (US) [66]	To evaluate feasibility of recruitment, retention, and intervention acceptability by intervention participants. Criteria: Not reported.	Lost to follow-up: nIG = 4 nCG = 2 (reasons: medical, lost interest) Final analysed: n = 64 nIG = 31 nCG = 33	Not reported.	Primary: GWG Secondary: GDM incidence Gestational HTN Preeclampsia Mode of delivery Neonatal outcomes Preterm delivery *	RCT IG: Integrating hope theory and goal-oriented episodic future thinking using a combination of weekly web intervention on Qualtrics to regulate stress management and 10 health coaching sessions bi-weekly to increase healthy eating and exercise. CG: Standard care.	Adherence: Web module: 25% completed at least 16/20 modules Health coaching: 71.4% attended 8–10 sessions Duration: 20 weeks	Age Ethnicity Marital status Education Employment Receiving public financial assistance Pre-pregnancy BMI Parity Age of youngest child Number of children Gestational age at enrolment
Quinlivan et al., 2011 (Australia) [67]	To evaluate whether a four-step multidisciplinary protocol of antenatal care for women with overweight and obesity would reduce the incidence of gestational diabetes. Criteria: WHO.	Lost to follow-up: n = 0 Final analysed: n = 124 nIG = 63 nCG = 61	A sample size of 126 women could detect a difference in gestational diabetes of 33% compared to 11% with a power of 80% and α error of 5%.	Primary: GDM incidence * (OR: 0.17; 95% CI: 0.03–0.95) Secondary: GWG * Neonatal BW	RCT IG: Four-step multidisciplinary model that includes continuity of care provider, weighing on arrival, brief dietary intervention by food technologist at every visit, and psychological assessment and intervention if needed. CG: Standard care.	Adherence: Not reported Duration: Not reported	Age Gravida Parity Race BMI category Plan for pregnancy Smoking status Drinking status Father involvement
Xu et al., 2022 (China) [68]	To evaluate the effect of an individualized weight management intervention during the second and third trimesters for pregnant women with excessive weight gain by observing gestational weight gain and perinatal outcomes. GDM: IADPSG.	Lost to follow-up: nIG = 10 nCG = 8 (transfer to another hospital) Analysed: n = 348 nIG = 203 nCG = 145	At least 176 in the intervention group and 117 in the control. A 95% power value used to detect a difference of −2.0 between the null hypothesis that both groups’ means were 15.0 and the alternative hypothesis that the mean of the control group was 17.0. The estimated group standard deviations were 5.0 and 4.0 for the intervention and control.	Primary: GWG * Secondary: GDM incidence * (RR: 0.33; 95% CI: 0.13–0.838) Mode of delivery Gestational HTN * Hospitalization stay Fetal distress *	RCT IG: Used social cognitive theory (SCT) to increase adherence to individual meal plans and exercise prescription. CG: Standard care.	Adherence: Not reported Duration: Not reported	Age Height Pre-pregnancy weight and BMI Gravidity Parity
Maleki et al., 2023 (Iran) [62]	To evaluate the effect of education through a mobile app on the health-promoting lifestyle of women with gestational diabetes. The secondary aim was to assess the effect of this intervention on blood sugar. Criteria: WHO.	Lost to follow-up: n = 0 Analysed: n = 76 nIG = 38 nCG = 38	Assuming α = 0.05, β = 95%, d = 11.14, s = 15.12, and 20% attrition, the final sample size calculated was 38 in each group.	Primary: Health-promoting lifestyle (HPLP) score * Secondary: FBG * PBG * (percent difference not provided)	RCT IG: App usage to increase education on GDM, nutrition and exercise. CG: Standard care.	Adherence: Not reported Duration: 4 weeks	Age Gestational age BMI Gravidity * Education Occupation * Planned pregnancy HPLP scores BGC
Premalatha et al., 2024 (India) [63]	To identify the effect of the Nutrition and Behavior Modification Program (NBMP) on maternal and neonatal outcomes of hyperglycemic mothers. Criteria: Not reported.	Lost to follow-up: nIG = 4 nCG = 7 (reason not mentioned) Analysed: n = 89 nIG = 45 nCG = 44	Not reported	Primary: BGC * (percent difference not provided) Secondary: GWG Mode of delivery NICU admission Mets/week *	RCT IG: One face-to-face education session on nutrition and exercise along with personalized, weekly WhatsApp messages on NMBP. CG: Standard care.	Adherence: Not reported Duration: From GDM diagnosis to delivery	Age Parity BMI Family history of diabetes GDM history BGC *
Banafshe et al., 2025 (Iran) [69]	To evaluate the effectiveness of a psychosocial-based intervention on weight management and pregnancy outcomes in pregnant women with overweight to obesity. Criteria: Not reported.	Lost to follow-up: nIG = 15 nCG = 11 (miscarriage or incomplete questionnaires) Analysed: n = 176 nIG = 86 nCG = 90	With a power of 80%, a significance level of 5%, and a 20% potential attrition rate, the required sample size was estimated at 202.	Primary and secondary not mentioned. GDM incidence Preeclampsia Induction Mode of delivery Agpar score NICU admission	RCT IG: Three educational sessions to increase knowledge about GWG, nutritional needs and exercise recommendations with ongoing support through text messages throughout pregnancy. CG: Standard care.	Adherence: Not reported Duration: From enrolment to delivery	Age Weight and BMI pre-pregnancy Education Spouse’s education Mother’s occupation Spouse’s occupation Ethnicity * Housing Income Previous deliveries Number of pregnancies Stillbirth history Number of living children Abortion history
Chan et al., 2018 (China) [70]	To compare the effectiveness of a lifestyle modification program (LMP) in early pregnancy with usual antenatal care in reducing GDM incidence; decreasing the proportion of infants born large-for-gestational-age (LGA) and being classified as macrosomia; and improving other maternal and birth outcomes. Criteria: WHO.	Lost to follow-up: nIG = 30 nCG = 24 (medical, drop-out) Analysed: n = 166 nIG = 80 nCG = 86	With a power of 90% at a 1% alpha level and two-sided test to detect an 83% reduction in the odds of decreased GDM in the intervention group when compared with the control group, assuming 30% lost to follow-up rate and 5% miscarriage rate, a final sample size of 110 participants per group was decided.	Primary: GDM incidence Secondary: LGA incidence Macrosomia Diet Mets/week *	RCT IG: LMP-Individualized diet and exercise plan given to each participant up to 24 weeks of gestation, where participants received bi-weekly face-to-face or phone consultations. CG: Standard care.	Adherence: IG: 14/80 (17.5%) Duration: 12 weeks	Age Gestational age Body weight Height BMI Pre-pregnancy weight and BMI Family history of diabetes GDM history Education level Occupation status Marital status **Monthly family income** * Parity Smoking Alcohol use
Mohsenzadeh-Ledari et al., 2022 (Iran) [71]	To examine the effects of a care intervention program on pregnancy outcome in pregnant women with Metabolic Syndrome during 2017–2018. Criteria: IDAPSG.	Lost to follow-up: nIG = 6 nCG = 5 (medical reasons) Analysed: n = 109 nIG = 54 nCG = 55	P1 = 0.3490, P2 = 0.6570, power = 0.9, α = 0.05, and n2/n1 = 1 was considered, and the required sample size in each group was estimated to be 60, with a drop-out rate of 15%.	Primary: GWG 2hPBG * (percent difference not provided) Secondary: GDM incidence * (OR: 3.59; 95% CI: 1.23–10.45) Hospitalization GDM nutritional diet prescription *	RCT IG: One motivational interview session, along with two nutrition consultations and three training sessions for pregnancy physical activity. CG: Standard care.	Adherence: Not reported Duration: Not reported	Age BMI Occupation Education * Economic status Weight * FBG HDL Triglycerides Gravidity Parity Abortion GDM
Zhu et al., 2025 (China) [64]	To explore the effect of interdisciplinary and diversified health education combined with personalized nutrition intervention on FPG, 2hPG, SDS, and SAS scores and pregnancy outcome in gestational diabetes mellitus (GDM). Criteria: Not reported.	Lost to follow-up: Not reported Analysed: n = 180 nIG = 90 nCG = 90	Not reported.	Primary: FBG * 2hPBG * HbA1c * (Percent difference not provided) Anxiety scale * Depression scale * Unfavourable pregnancy outcomes *	RCT IG: Interdisciplinary and diversified health education combined with personalized diet intervention (duration not reported). CG: Standard care.	Adherence Not reported Duration: Not reported	Age Gestational age HbA1c PBG HbA1c SAS SDS
Wang et al., 2023 (China) [65]	To investigate the impact of evidence-based care on glucose levels and pregnancy outcomes in patients with gestational diabetes mellitus. Criteria: ADA and IADPSG.	Lost to follow-up: Not reported Analysed: n = 120 nIG = 60 nCG = 60	Not reported.	Primary and Secondary not mentioned. FBG 2hPBG HbA1c * (percent difference not provided) Premature labour Gestational HTN * Macrosomia *	RCT IG: Continuous, evidence-based care involving formation of a care team and intervention for identified challenges along with nutrition and exercise guidance throughout pregnancy. CG: Non-continuous, nutrition and exercise education.	Adherence: Not reported Duration: From GDM diagnosis to delivery	Age BMI Parity
Koivusalo et al., 2015 (Finland) [72]	To assess whether gestational diabetes mellitus (GDM) can be prevented by a moderate lifestyle intervention in pregnant women who are at high risk for the disease. Criteria: ADA.	Lost to follow-up: nIG = 4 nCG = 7 (Reasons: miscarriage, other) Analysed: n = 269 nIG = 144 nCG = 125	A sample of ∼280 pregnant women (140 in each group) was required to detect differences in the incidence of GDM between the intervention (20%) and control (35%) groups of 15% (α = 0.05, power = 80%). Assumed a 40% drop-out rate.	Primary: GDM incidence * (13.9%; CI 95%: 8.7–20.6%) Secondary: FBG * 2hPBG * (Percent difference not provided) GWG * Preeclampsia Gestational HTN Preeclampsia Mode of delivery Neonatal outcomes	RCT IG: Individualized nutrition and exercise lifestyle counselling according to stage of pregnancy. CG: Standard care.	Adherence: Not reported Duration: From study entry to delivery	Age Weight and BMI pre-pregnancy and at baseline Height Gestational age Educational status Previous deliveries Prior GDM Cholesterol HDL 2hPBG HbA1c Insulin HOMA-IR Smoking Alcohol use Physical activity

* = Statistically significant difference between-group comparisons; IG = intervention group; CG = control group.

## Data Availability

No new data were created or analysed in this study. Data sharing is not applicable to this article.

## Abbreviations

The following abbreviations are used in this manuscript:

GDM	Gestational Diabetes Mellitus
LGI	Low Glycemic Index
MNT	Medical Nutrition Therapy
GI	Glycemic Index
RCT	Randomized Controlled Trial
BG	Blood Glucose
HGI	High Glycemic Index
GWG	Gestational Weight Gain
PBG	Postprandial Blood Glucose
FBG	Fasting Blood Glucose
